# Helpers don’t help when it’s hot in a cooperatively breeding bird, the Southern Pied Babbler

**DOI:** 10.1093/beheco/arad023

**Published:** 2023-04-15

**Authors:** Amanda R Bourne, Amanda R Ridley, Susan J Cunningham

**Affiliations:** FitzPatrick Institute of African Ornithology, DST-NRF Centre of Excellence, University of Cape Town, Private Bag X3, Rondebosch 7701, South Africa; Australian Wildlife Conservancy, 322 Hay Street, Subiaco 6008, Western Australia; FitzPatrick Institute of African Ornithology, DST-NRF Centre of Excellence, University of Cape Town, Private Bag X3, Rondebosch 7701, South Africa; Centre for Evolutionary Biology, School of Biological Sciences, University of Western Australia, Crawley 6009, Australia; FitzPatrick Institute of African Ornithology, DST-NRF Centre of Excellence, University of Cape Town, Private Bag X3, Rondebosch 7701, South Africa

**Keywords:** arid zone, avian reproduction, cooperative breeding, dryland ecology, load-lightening, reproductive success, temporal variability hypothesis, *Turdoides bicolor*

## Abstract

Cooperative breeding, where more than two individuals invest in rearing a single brood, occurs in many bird species globally and often contributes to improved breeding outcomes. However, high temperatures are associated with poor breeding outcomes in many species, including cooperative species. We used data collected over three austral summer breeding seasons to investigate the contribution that helpers make to daytime incubation in a cooperatively breeding species, the Southern Pied Babbler *Turdoides bicolor*, and the ways in which their contribution is influenced by temperature. Helpers spent a significantly higher percentage of their time foraging (41.8 ± 13.7%) and a significantly lower percentage of their time incubating (18.5 ± 18.8%) than members of the breeding pair (31.3 ± 11% foraging and 37.4 ± 15.7% incubating). In groups with only one helper, the helper’s contribution to incubation was similar to that of breeders. However, helpers in larger groups contributed less to incubation, individually, with some individuals investing no time in incubation on a given observation day. Helpers significantly decrease their investment in incubation on hot days (>35.5°C), while breeders tend to maintain incubation effort as temperatures increase. Our results demonstrate that pied babblers share the workload of incubation unequally between breeders and helpers, and this inequity is more pronounced during hot weather. These results may help to explain why recent studies have found that larger group size does not buffer against the impacts of high temperatures in this and other cooperatively breeding species.

## Teaser

In cooperatively breeding Southern Pied Babblers, non-breeding helpers reduce their incubation effort on hot days, but breeders do not. The significant decline in helper contribution to incubation at high temperatures could help to explain why previous research has shown that larger pied babbler groups do not have higher breeding success than smaller groups during hot weather.

## INTRODUCTION

Cooperative breeding, where more than two individuals invest in rearing a single brood (Cockburn 2002), occurs in ~9% of bird species globally (Cockburn 2006; Riehl 2013). In these species, helpers care for young that may not be their own (Cockburn 2002) and the survival of young often improves with increasing group size (Canestrari, Marcos, et al. 2008; Ridley 2016; Bourne et al. 2020a). The investment that helpers make in young may be additive to that of parents, increasing the overall amount of care provided to young (Canestrari, Chiarati, et al. 2008; Pike et al. 2019), or compensatory, enabling parents to reduce their investment while young still receive a similar amount of care overall (Hatchwell 1999; Savage et al. 2015; van Boheemen et al. 2019), a phenomenon known as “load-lightening” (Crick 1992; Meade et al. 2010; Mumme et al. 2015; Langmore et al. 2016). Global comparative studies have shown that the distribution of cooperatively breeding (Rubenstein and Lovette 2007; Jetz and Rubenstein 2011; Lukas and Clutton-Brock 2017; Shen et al. 2017) and group-living (Griesser et al. 2017; Firman et al. 2020) species is associated with harsh environments characterized by high spatial and temporal variability in rainfall, such as arid and semi-arid systems (although see Gonzalez et al. 2013; Lin et al. 2019). This association suggests that the contribution made by additional group members may buffer against environmental uncertainty (Rubenstein and Lovette 2007; Jetz and Rubenstein 2011; Russell 2016; Cornwallis et al. 2017; Lukas and Clutton-Brock 2017; Shen et al. 2017), at least up to an optimal group size (Markham et al. 2015; Ridley 2016). The buffering effect of cooperation under conditions of environmental uncertainty assumes that the influence of helpers on reproductive success varies with environmental conditions and becomes more important as conditions worsen (Hatchwell 1999; Langmore et al. 2016). Several recent studies have empirically tested the benefits of cooperation for reproduction across varying environmental conditions (e.g., Covas et al., 2008; Langmore et al., 2016; Bourne et al., 2020c, 2020b; Guindre-Parker and Rubenstein, 2020; van de Ven, Fuller, et al., 2020; Riehl and Smart, 2022), looking for interactions between measures of cooperation, such as group size or the number of helpers present, and challenging environmental conditions such as low rainfall or high temperatures.

To date, the evidence for a buffering benefit of cooperation to environmental conditions is mixed. Recent research on Southern Pied Babblers *Turdoides bicolor* (hereafter “pied babblers”) has shown that larger group sizes do not buffer against the negative impacts of high temperatures or low rainfall on reproductive success (Bourne et al., 2020c, 2020b, 2020a; Bourne, Ridley, McKechnie, et al., 2021b; reviewed in Ridley et al., 2021). Similarly, recent empirical studies have not detected a buffering effect of larger group size in Superb Starlings *Lamprotornis superbus* (Guindre-Parker and Rubenstein 2020), Sociable Weavers *Philetairus* socius (D’Amelio et al. 2022), Greater Ani *Crotophaga major* (Riehl and Smart 2022), Seychelles Warblers *Acrocephalus sechellensis* (Borger et al. 2022), or Meerkats *Suricatta suricatta* (van de Ven, Fuller, et al. 2020). However, there is empirical evidence in support of a buffering effect of larger group size in some studies, for example, in a concurrent study on Meerkats (Groenewoud and Clutton-Brock 2021) and recent research on White-browed Sparrow-weavers *Plocepasser mahali* (Capilla-Lasheras et al. 2021).

The costs of parental care incurred by both breeders and helpers during the course of a breeding attempt (Harshman and Zera 2007; Blomberg et al. 2013; Guindre-Parker and Rubenstein 2018) could provide a mechanism to explain why buffering effects of larger group size were not observed in some species. Incubation is costly in temperate environments where eggs need to be kept warm (Ardia et al. 2010;

Nord et al. 2010; Nord and Cooper 2020), but also extremely challenging in warm environments (Amat and Masero 2004; Coe et al. 2015; Nwaogu et al. 2017), where incubating birds must prevent eggs from overheating (Grant, 1982; Carroll et al. 2015; McDonald and Schwanz 2018) while also thermoregulating themselves (O’Connor et al. 2018; DuRant et al. 2019; McKechnie 2019). Individuals can, and do, make adjustments to their levels of investment in breeding and/or helping in order to minimize these costs (Heinsohn 2004; Canestrari et al. 2007; Wiley and Ridley 2016; Bambini et al. 2019; van de Ven, McKechnie, et al. 2020; Cunningham et al. 2021; Rotics and Clutton-Brock 2021; Sharpe et al. 2021). As incubating birds reach limits in their ability to tolerate high temperatures over long periods, they undertake more frequent or longer incubation recesses (Clauser and McRae 2017) and may ultimately abandon their nests (Sharpe, Cale and Gardner, 2019). Breeders may be expected to continue to invest in young as the risk to themselves increases (Ghalambor and Martin 2001; Langmore et al. 2016), while helpers may be expected to invest less than either member of the breeding pair, particularly during unfavorable environmental conditions (Reyer 1984; Canestrari, Chiarati, et al. 2008; Rotics and Clutton-Brock 2021). One reason for this could be that helpers prefer to maintain their own body condition when the group attempts to breed during adverse conditions, because they are likely to have higher reproductive success later in life, when they become a breeder (McNamara et al. 2009; Raihani et al. 2010).

Pied babblers provide a useful model for exploring why recent studies have found that larger group sizes do not buffer against the impacts of environmental variability in some cooperatively breeding species. Pied babblers are cooperative breeders that occupy year-round territories in an arid environment characterized by challenging environmental conditions including high summer temperatures and highly variable rainfall (MacKellar et al. 2014; Ridley 2016; van Wilgen et al. 2016). Past research on this study population in the southern Kalahari means that much is known about their cooperative behaviors (Ridley 2016), reproductive outcomes (Bourne et al. 2020c), and the effect of high temperatures and drought in this species (reviewed in Ridley et al. 2021). High air temperatures influence foraging efficiency and body mass maintenance (du Plessis et al. 2012), incubation behavior (Bourne, Ridley, McKechnie, et al. 2021b), nestling size (Cunningham, et al. 2021), daily growth rates (Bourne, Ridley, Spottiswoode, et al. 2021c), provisioning behavior (Wiley and Ridley 2016; Bourne, Ridley, Spottiswoode, et al. 2021c), recruitment into the adult population (Bourne et al. 2020b), long-term population viability (Conradie et al. 2019; Ridley et al. 2021), and physiological responses (Bourne, Ridley, McKechnie, et al. 2021b; Moagi et al. 2021). In good seasons, pied babbler groups can fledge multiple broods (Ridley and Raihani 2008). Groups typically incubate clutches constantly throughout the day (median = 99% of the time during the day), except on days when air temperatures exceed 37°C (Bourne, Ridley, McKechnie, et al. 2021b). Nest attendance declines when air temperatures exceed 37°C, and nests are abandoned after consecutive hot days (Bourne, Ridley, McKechnie, et al. 2021b). The probability of clutches hatching is halved at air temperatures above 35.3°C (Bourne et al. 2020c; Bourne, Ridley, McKechnie, et al. 2021b). No successful breeding has been observed in pied babblers when maximum daily air temperatures exceed 38°C on average during the breeding attempt (Bourne et al. 2020c).

Here, we explore the question of whether breeders (who are the genetic parents of young raised in the single clutch per group in >95% of cases, Nelson-Flower et al. 2011) contribute more during incubation, particularly under unfavorable conditions, than helpers in the cooperative group. Specifically, we use detailed incubation behavior data collected over three austral summer breeding seasons to investigate the relative contributions that breeders and helpers make during incubation in the cooperatively breeding pied babbler, and the ways in which their respective contributions are influenced by daily maximum air temperature (*T*_max_). We tested for the influence of interactions between air temperature and rank (breeder/helper), number of helpers in the group (continuous numeric) and rank and, for helpers only, air temperature and the number of helpers in the group on the proportion of time that individual birds spent incubating per observation day. We hypothesized that breeders would be more likely to maintain incubation effort at higher air temperatures, whereas helpers would be more likely to reduce incubation effort at higher air temperatures. These analyses help to explain why a larger group size does not appear to buffer against adverse environmental conditions in this species (Bourne et al. 2020b, 2020c).

## MATERIALS AND METHODS

### Study site and system

Data were collected for each austral summer breeding season between September 2016 and February 2019 (three breeding seasons in total) at the Kuruman River Reserve (33 km^2^, KRR; 26°58’S, 21°49’E) in the southern Kalahari. Mean summer daily maximum air temperatures in the region average 34.5 ± 1.4°C and mean annual precipitation averages 174.0 ± 70.1 mm (2005–2020, Bourne et al. 2020b). The Kalahari region is characterized by hot summers and periodic droughts (van Wilgen et al. 2016), with extremely variable rainfall between years (MacKellar et al. 2014).

Pied babblers are medium-sized, sexually monomorphic passerines (Bourne, Cunningham, et al. 2021) and live in cooperative groups year-round, ranging in size from 3–15 adults (Raihani and Ridley 2007). They nest in open-cup grass nests and breed during the austral summer (September–March, Bourne et al. 2020b). Pied babbler groups consist of a single breeding pair (“dominant” birds), subordinate adult helpers of both sexes and juveniles (individuals < 1-year-old; Ridley 2016). Pied babbler breeding pairs are highly monogamous (Nelson-Flower et al. 2011). Helpers are usually, but not always, the offspring or sibling of at least one member of the breeding pair. Even when a helper is unrelated to an opposite-sex member of the breeding pair, extrapair offspring are rare and account for <5% of all reproduction (Nelson-Flower et al. 2011). All adult group members engage in cooperative behaviors including territory defense and care of young (Ridley and Raihani 2007; Ridley 2016). Pied babblers lay clutches of ~3 eggs (Ridley 2016), which hatch after 14 ± 1.2 days (Bourne et al. 2020c), and nestlings fledge after 15.4 ± 1.7 days (Bourne et al. 2020c). Incubation is typically almost constant throughout the day (Bourne, Ridley, McKechnie, et al. 2021b), to regulate egg temperature (White and Kinney 1974; Grant 1982). Birds in the study population are uniquely identifiable by metal and color rings fitted to their legs as nestlings and are habituated to human observation within distances of 1 to 5 m (Ridley 2016; Ridley et al. 2021), enabling detailed observations under natural conditions, without causing a disturbance or modifying behavior. Breeders can be identified through their behavior. For example, only the breeding female incubates overnight (Ridley 2016) and breeding pairs have distinctive duets (Wiley and Ridley 2018).

### Adult behavior data

To understand the different contributions that breeders and helpers make to incubation, we collected data on the duration of all daytime incubation bouts per individual by waiting near the nest at first light, observing the first bird to replace the breeding female in the morning (05h00 – 06h28, 4 min before sunrise on average [range 36 min before to 26 min after sunrise]) and remaining with the group all day until 19h00 (46 observation days at 35 nests, also see Bourne, Ridley, McKechnie, et al. 2021b). We recorded the start and end time of each incubation bout and the identity of each incubating bird. Incubation was defined as the observed bird settling onto the nest, covering the clutch with its body. Incubation bouts that lasted less than 60 s were not included. Nest attendance behaviors that involved perching on the edge of the nest or on branches next to the nest were not considered incubation. For most nests (*n* = 28), observations were collected once (whole-day observation) during the incubation period and on two or more days (up to a maximum of 4 days) for the remaining seven nests, depending on the number of active nests being monitored at the time. The limited number of breeding attempts for which we were able to collect multiple days of observations meant that each observation day was treated as independent in the analyses, and the repeatability of observed patterns of individual incubation behavior could not be tested. Incubation bout data were used to calculate the proportion of time per day that each group member incubated the clutch. Our focus on the daytime incubation effort enabled consideration of the effects of high temperatures on incubation behavior. However, as the breeding female also incubates overnight, our approach necessarily underestimates the breeding female’s total contribution to incubation.

We additionally conducted 20-min continuous time-activity focal behavior observations (Altmann 1974) on up to three different adult birds per day. Each bird was observed during a minimum of six and a maximum of 27 focal sessions per observation day (Bourne, Ridley, Spottiswoode, et al. 2021c). We collected focal behavior data from 44 different individual birds in 15 different groups incubating clutches at 40 different nests. While pied babblers invest in a single nest at a time, we observed multiple breeding attempts by the same groups over multiple breeding seasons. Group sizes ranged from three to six adults, including the breeding pair and one to four adult helpers. The number of adults present remained constant within all breeding attempts but varied between breeding attempts and/or breeding seasons in seven of the 15 groups. We, therefore, collected data from seven groups with one helper, eight groups with two helpers, four groups with three helpers and five groups with four helpers over three breeding seasons. Sub-adult individuals (from recent, dependent fledglings up to 1 year of age) were present on approximately half of the observation days (*n* = 21). Of the groups with sub-adults present, sub-adults represented 42% of non-breeding group members on average (range 16–75%). Sub-adults rarely invest in helping behavior and thus, for the purposes of this study, were not considered helpers and were not included in the analyses relating to the number of helpers in a group.

Focal sessions (involving multiple 20-min focal observations) lasted 2 h each, with the first starting at 07h00 and the last starting at 17h00, as described in detail by Bourne et al. (2021c). We collected 1,291 focal behavior observations (mean focal length = 19.4 ± 0.99 min) over 64 focal days (mean daily observation length over a focal day = 411 ± 101 min) during three austral summer breeding seasons (*n* = 29 days between 14 October 2016 and 18 March 2017, *n* = 16 days between 22 September 2017 and 7 January 2018, *n* = 19 days between 14 October 2018 and 18 December 2019). Behavior observations were collected across a range of naturally occurring daily maximum air temperatures, on 33 hotter focal days (*T*_max_ ≥ 35.5°C, range 35.6–40.8°C) and 31 cooler focal days (*T*_max_ < 35.5°C, range 20.7–35.2°C). All group sizes were sampled across a range of temperatures. The threshold temperature, 35.5°C, is taken from previous studies which identified a change in foraging efficiency (du Plessis et al. 2012), provisioning effort (Wiley and Ridley 2016), and hatching success (Bourne et al. 2020c) at *T*_max_ ≥ 35.5°C in this species. On the 64 observation days, we observed 31 males and 33 females, 32 breeders and 32 helpers. All birds for which we recorded fewer than six focal observations on an observation day were removed from the analyses (*n* = 2 individuals). Behavior data were captured on a Mobicel smartphone using Prim8 software (McDonald and Johnson 2014), in which the duration of each observed behavior can be recorded to the nearest second.

For analyses of time budgets, we summed the amount of time each individual was observed foraging (foraging effort, including searching for and handling prey), attending the nest (incubation as described above; shading and incubating were not distinguished due to limited visibility of birds once settled into their nests), resting (preening, standing, and perching) and engaging in other activities (e.g., hopping along the ground, flying, being vigilant and interacting with neighboring groups) and calculated the proportion of time individual birds allocated to each set of activities across all focals per individual per observation day, following Bourne et al. (2021c).

### Weather data

Daily maximum air temperature (*T*_max_, in °C) and rainfall (mm) data were collected from an on-site weather station at the KRR (Vantage Pro2, Davis Instruments, Hayward, CA, USA). *T*_max_ on the measurement day was used to ensure consistency with other studies in a similar environment (Cunningham et al. 2013; Wiley and Ridley 2016; van de Ven, McKechnie, et al. 2020; Bourne, Ridley, Spottiswoode, et al. 2021c). Rainfall was summed for each breeding season (September to March) in order to distinguish between relatively wetter and drier breeding seasons.

## STATISTICAL ANALYSES

Statistical analyses were conducted in the R statistical environment, v 3.6.0, using R Studio (R Core Team 2020). All continuous explanatory variables were scaled by centering and standardizing by the mean (Schielzeth 2010; Harrison et al. 2018). All explanatory variables were tested for correlation with one another (Fox and Monette 1992) and there was no evidence of multicollinearity (all VIF < 1.9).

To determine which variables best predicted the proportion of time adults spent foraging, resting, and attending the nest, we fitted binomial GLMMs with Penalised Quasi-Likelihood (glmmPQL) in the R package *MASS* (Venables and Ripley 2002). The glmmPQL approach, which precludes model selection (Bolker et al. 2009), was used to address overdispersion in the data not adequately resolved by the inclusion of an observation-level random term, while still allowing the inclusion of the random term for group identity and nest identity. The proportion of time individuals spent on each activity was modeled as a combined vector of total time spent on the selected activity (seconds) versus the total time observed (seconds). The models included predictor variables *T*_max_, season and number of helpers in the group, as well as the sex and rank of the focal bird.

Interactions of interest (rank*T_max_, number of helpers*rank and, for helpers only, number of helpers*T_max_) were tested in post-hoc generalized linear mixed effects models (GLMMs) with a binomial distribution and a logit link function in the package lme4 (Bates et al. 2015). The model for the number of helpers*T_max_ used a subset of the data excluding breeders in order to directly test whether helpers adjusted their incubation behavior in response to temperature differently depending on the number of other helpers in the group. Previous work on this species has found no sex-specific differences in day-time incubation and other cooperative behaviors (Bourne et al. 2021a, 2021b, 2021c). We considered the influence of each interaction term on the proportion of focalled time spent incubating, with group identity and nest identity included as random terms in all analyses. The inclusion of bird identity as a random term in addition to nest identity and group identity resulted in unstable models and, of the three random terms, nest identity and group identity explained the greatest proportion of variation while avoiding destabilizing the models (Grueber et al. 2011; Harrison et al. 2018).

## RESULTS

Maximum air temperatures on data collection days ranged from 20.7°C to 40.8°C (mean = 34.2 ± 4.5°C). The 2016/17 breeding season was wetter (226.6 mm) than the breeding seasons in 2017/18 (165.2 mm) and 2018/19 (88.4 mm). The number of adult helpers in a group, in addition to the breeding pair, averaged 2 ± 1 adults (range: 1–4).

Individual incubation bouts were variable in length (*n* = 437; mean = 80.1 ± 76.4 min; range: 1–420 min). Approximately half of the individual incubation bouts lasted <60 min (*n* = 235). The average duration of incubation bouts did not differ significantly between hotter days (≥ 35.5°C; mean = 87.3 ± 88.4 min; range: 1–420 min) and cooler days (< 35.5°C; 73.8 ± 63.6 min; range: 1–302 min; Wilcoxon rank sum *W* = 24562, *P* = 0.546). Total incubation effort as time spent attending the nest in minutes per adult individual per day averaged 171 ± 138 min (range 0–534 min). Observation time ranged from 12 to 14 h per day (mean = 13.4 ± 0.4 h), during which time most nests were incubated constantly.

Sub-adult individuals (< 1-year-old) generally incubated for very brief intervals (*n* = 18 incubation bouts; mean duration = 18.4 ± 15.4 min, range: 2–54 min), and for relatively short periods in total (*n* = 8 observation days; mean duration = 23.5 ± 17.4 min, range 3–54 min; excludes one sub-adult individual from a group of three adults that incubated for 120 min in total across the observation day). All analyses, therefore, exclude data collected on sub-adults. In 93% of groups with one helper, all adult group members incubated on every observation day (*n* = 14 observation days). All adults incubated every observation day in only 70% of groups with two helpers (*n* = 17 observation days), 60% of groups with three helpers (*n* = 5), and zero groups with four helpers (*n* = 11). Both members of the breeding pair incubated at every nest on every observation day, and for at least an hour every day (range: 55–535 min; mean = 251 ± 107 minutes) compared with helpers, who generally contributed less to incubation (range: 0–521 min; mean = 104 ± 124 min). In groups with four helpers, we recorded individual helpers that did not incubate at all on 67% of observation days.

**Figure 1:**
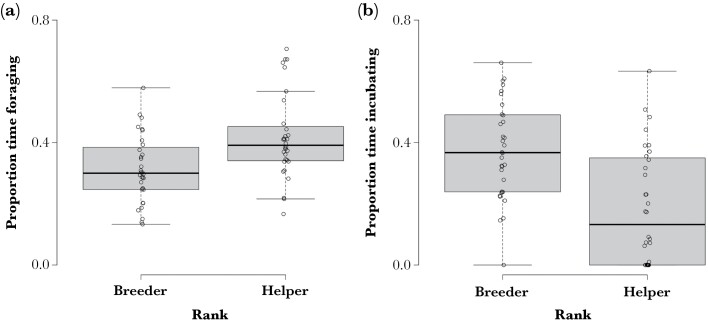
Proportion of time spent foraging (a) and incubating at the nest (b) differed between breeder and helper Southern Pied Babblers Turdoides bicolor. Data from 64 days of focal observations on 44 different individuals from 40 nests by 15 groups over three breeding seasons.

Pied babblers in incubating groups spent 36.5 ± 13.4% time foraging, 26.5 ± 12% time resting, 28 ± 19.7% time incubating, and the remaining time engaged in other activities. Helpers spent significantly more time foraging (41.8 ± 13.7%) and significantly less time incubating (18.5 ± 18.8%) than breeders (31.3 ± 11%; 37.4 ± 15.7%; Table 1, Figure 1). Birds also spent significantly more time foraging (44.6 ± 15.3%) and less time incubating (23.8 ± 20.9%) overall during the 2017/18 breeding season than in the wetter 2016/17 breeding season (32.3 ± 13.3%; 30.9 ± 20.7%; Table 1). Time spent foraging (36.1 ± 8.6%) and incubating (26.9 ± 17%) in the driest 2018/19 breeding season did not differ significantly from either of the preceding years. The proportion of time spent resting did not vary significantly by any of the predictors included in the model (Table 1).

**Table 1:** glmmPQL model outputs for factors influencing the proportion of time spent (a) foraging, (b) resting, and (c) attending the nest

Model	Parameters	Estimate	SE	*t*-value	*P*-value
Proportion time spent foraging	*Intercept*	−0.969	0.128	−7.554	0.000
	*T*_max_	0.112	0.068	1.642	0.116
	**Rank (Helper)**	**0.407**	**0.116**	**3.496**	**0.002**
	Sex (Male)	0.033	0.119	0.277	0.785
	Number of helpers	0.032	0.073	0.446	0.658
	Season				
	**Season (2017/18)**	**0.605**	**0.167**	**3.629**	**0.001**
	Season (2018/19)	0.098	0.160	0.614	0.543
Proportion time spent resting	*Intercept*	−1.171	0.210	−5.568	0.000
	*T*_max_	0.128	0.165	1.195	0.255
	Rank (Helper)	0.219	0.165	1.324	0.210
	Sex (Male)	0.142	0.167	0.852	0.411
	Number of helpers	0.111	0.112	0.994	0.329
	Season				
	Season (2017/18)	−0.127	0.275	−0.462	0.647
	Season (2018/19)	0.012	0.263	0.044	0.965
Proportion time spent attending the nest	*Intercept*	−0.189	0.235	−0.803	0.427
	*T*_max_	−0.249	0.134	−1.860	0.077
	**Rank (Helper)**	−**1.027**	**0.224**	−**4.595**	**0.000**
	Sex (Male)	−0.357	0.222	−1.604	0.124
	Number of helpers	−0.195	0.150	−1.302	0.201
	Season				
	**Season (2017/18)**	−**0.757**	**0.362**	−**2.089**	**0.044**
	Season (2018/19)	0.047	0.322	0.147	0.884

Models fitted to data from 64 days of focal observations on 44 different individuals from 40 nests by 15 groups over three breeding seasons. Reference level for the categorical term Rank = Breeder; reference level for the categorical term Sex = Female; reference level for categorical term Season = 2016/17. Significant terms are shown in bold.

**Figure 2:**
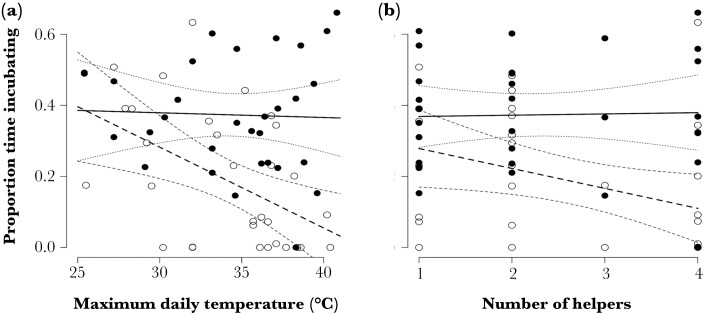
Proportion of time that individual Southern Pied Babblers Turdoides bicolor spent incubating in relation to maximum daily air temperature and rank (a) and number of helpers in the group and rank (b). Breeders = filled circles and solid model fit line, helpers = open circles and dashed model fit line. Confidence intervals shown as dotted lines in all cases. Data from 65 days of focal observations on 46 different individuals from 40 nests by 15 groups over three breeding seasons.

Rank interacted with temperature (GLMM Est = −0.118 ± 0.009, *z* = −13.742, *P* < 0.001) and the number of helpers in the group (GLMM Est = −0.164 ± 0.007, *z* = −23.679, *P* < 0.001). Helpers decreased the proportion of time they spent incubating as temperatures increased, to zero at very high temperatures for some individuals, whereas breeders maintained the proportion of time spent incubating at high temperatures (Figure 2a). Breeders spent a fairly consistent ~30% of focal time incubating across all group sizes, but the contribution of helpers varied widely relative to the number of helpers in the group, from 25.9 ± 20.9% in groups with one helper to only 4.5 ± 9.2% time incubating on average per individual in groups with four helpers (Figure 2b). After sub-setting the data to helpers only, to avoid confounding group size and rank, there was no significant interaction between the number of helpers in the group and temperature (GLMM Est = −8.188 ± 5.115, *z* = −1.601, *P* = 0.109), with all helpers tending to decrease incubation effort as temperatures increased.

## DISCUSSION

We investigated the influence of *T*_max_, rank and the number of helpers in the group on incubation effort in pied babblers, a cooperatively breeding species known to engage in load-lightening behaviors (Raihani and Ridley 2008; Ridley and Raihani 2008; Wiley and Ridley 2016). High temperatures negatively affect reproductive success in many bird species (Cunningham et al. 2013; Sharpe et al. 2019, 2021; van de Ven, McKechnie, et al. 2020; Corregidor-Castro and Jones 2021), including pied babblers (Bourne et al. 2020c). In cooperatively breeding pied babbers, the breeding pair undertake most incubation (Bourne et al. 2021b). In addition to the breeding female incubating overnight, we show that both members of the pair incubate every day, often for extended periods. Helpers also invest in incubation. However, particularly in groups with more than one helper, helpers typically spend significantly more time foraging and significantly less time incubating (about half the amount of time on average) than members of the breeding pair. At high temperatures, helpers significantly decreased their incubation effort. Investment in incubation by helpers often reduced to 0% of observation time at very high temperatures, while breeders maintained incubation effort at approximately 20–30% of observation time as temperatures increased. This is in contrast to previous findings in this species, where breeder investment in the provisioning of nestlings declined at high temperatures, but helper investment did not (Wiley and Ridley 2016). One possible explanation for this discrepancy is a difference in the benefits helpers versus breeders attain at different stages of the breeding cycle. Hatching probabilities are lower than fledgling survival probabilities (Bourne et al. 2020c) and breeders face a risk of oocide (egg-breaking) from competing females (Nelson-Flower et al. 2013). Therefore, breeders may be more motivated than helpers to invest in young during this stage, to increase the probability of reproductive success. At later stages, such as the nestling or fledgling stage, breeders may prefer to initiate a new breeding attempt rather than continue to invest in older young that are being provisioned by other group members. This pattern of behavior has been observed in pied babblers (Ridley and Raihani 2008), Western Australian magpies *Gymnorhina tibicen dorsalis* (Pike et al. 2019) and superb fairy-wrens *Malurus cyaneus* (Russell et al. 2007)Russell et al. 2008). Helpers may prefer to invest in older young, that are more likely to survive than eggs (Ridley and van den Heuvel 2012), since this increases their likelihood of gaining benefits either directly via group augmentation(Kokko et al. 2001) or indirectly via fitness benefits if they are related to the young.

Helper investment was strongly influenced by the number of helpers present in the group, whereas breeders maintained incubation effort regardless of the number of helpers available. In groups with one helper, the single helper made a similar contribution to the breeding pair in terms of time invested in incubation. However, helpers in larger groups invested a significantly lower proportion of their time in incubating on average. In this species, small groups are significantly more likely to go extinct than large groups (Ridley et al. 2022). Specifically, small groups that do not successfully breed have an elevated risk of extinction before the next breeding season (Ridley et al. 2022). Therefore helpers in smaller groups may invest more in incubation compared to helpers in large groups due to the greater potential risk of group extinction if there is no reproductive success. All helpers, regardless of the number of helpers available, significantly decreased their investment in incubation as temperatures increased. Helpers may prefer to maintain their own body condition during very hot conditions, prioritizing their own survival over that of young that are not their own, because helpers in this species have the possibility of gaining a breeding position later in life (Nelson-Flower et al. 2018).

The presence of helpers is very important for reproductive success in pied babblers (Bourne et al. 2020a) and pied babblers rarely breed successfully as a pair (Ridley 2016). However, in contrast to the nestling provisioning stage (Wiley and Ridley 2016), breeders did not benefit from increased load-lightening in groups with more helpers during the incubation stage. Rather, helpers either shared the work with each other or some helpers contributed more than others. Our finding that pied babbler helpers reduce their investment in incubation before breeders do suggests that load-lightening benefits of larger group size, whereby each individual can invest less in the task at hand (incubation) and more in their own self-maintenance (foraging, resting), see Crick (1992), Meade et al. (2010), and Ridley (2016), accrue mainly to helpers during the incubation stage of a cooperative breeding attempt in this species.

Several recent empirical studies (Guindre-Parker and Rubenstein 2020; Capilla-Lasheras et al. 2021; Groenewoud and Clutton-Brock 2021; D’Amelio et al. 2022; Riehl and Smart 2022), including studies of this species, the pied babbler (Bourne et al. 2020b, 2020c), have sought to explain the poorly understood association between cooperative behavior and highly variable environments in mammals and birds (Rubenstein and Lovette 2007; Cockburn and Russell 2011; Rubenstein 2011; Cornwallis et al. 2017; Lukas and Clutton-Brock 2017; Lin et al. 2019; Downing et al. 2020; Firman et al. 2020). These studies test the influence of group size (or a similar measure of helping behavior) on the effect that harsh environmental conditions (e.g., high temperatures, drought) have on reproduction and survival in cooperatively breeding species in the wild. Despite the expectation that cooperative breeding would buffer individuals against the impact of harsher or more variable environmental conditions, we have consistently found no evidence for a buffering effect of group size on any life history parameter in pied babblers, including survival (Bourne et al. 2020b), reproduction (Bourne et al. 2020c), parental care behavior (Bourne, Ridley, McKechnie, et al. 2021b; Bourne, Ridley, Spottiswoode, et al. 2021c), physiological responses (Bourne, Ridley, McKechnie, et al. 2021b), or body mass change (Bourne et al. 2020b; Bourne, Ridley, McKechnie, et al. 2021b;Bourne, Ridley, Spottiswoode, et al. 2021c). This was despite considerable statistical power to detect interactions should they have been present, particularly for the long-term data presented by Bourne et al. (2020c) and Bourne et al. (2020b). Our finding that pied babbler helpers contribute much less when hot conditions are experienced during incubation could help to explain why we have consistently found that larger pied babbler groups do not have higher breeding success than smaller groups during hot weather. A recent paper by Cockburn (2020) suggests that the apparent relationship between cooperative breeding and temporal variability may be the result of geographical and habitat biases in sampling efforts. If this is the case, buffering effects of larger group size may not, in fact, be expected.

Contributions to incubation by helpers do not increase with group size during high temperatures in this species, but instead decline. Future research could explore these patterns further by assessing whether individual incubation behaviors were repeatable across multiple days within the same breeding attempt. In addition, helper contribution may be affected by how soon the helper intends to disperse from the group, with previous research on pied babblers indicating that dispersal is costly (Ridley et al. 2008) and therefore individuals that are about to disperse provide significantly less help than other group members (Ridley 2016). Here, we present evidence that pied babblers share the workload of incubation unequally between breeders and helpers, particularly in larger groups and at high temperatures. Specifically, in groups with only one helper, the helper’s contribution to incubation was similar to that of the male breeders. In larger groups, we demonstrate clearly that helpers decrease incubation effort on hotter days whereas breeders do not. Helper contributions to incubation in groups with more than one helper dropped to zero in some cases on very hot days (>35.5°C). This may represent the classical cost-benefit trade-off as described by Ghalambor and Martin (2001), in which those adults with a greater likelihood of surviving to breed again are more likely to favor their own survival over that of the young when faced with challenging conditions. In this case, helpers, who are usually younger than breeders, have a greater likelihood of surviving to gain a breeding position in the future if they terminate care in the brood before temperatures increase to a point that imposes a physiological cost on the helper. In contrast, breeders, who are normally older than helpers, may be willing to take more risks to ensure the survival of the eggs. Our observations contribute towards explaining why the hypothesized buffering effects of larger group sizes have not been detected in pied babblers and some other arid-zone species despite considerable theoretical support for the temporal variability hypothesis, and some empirical evidence from other species in southern Africa.

## Notes

We thank the Kuruman River Reserve (KRR) and surrounding farms, Van Zylsrus, South Africa, for access to the land on which this study was conducted and the Fitzpatrick Institute of African Ornithology for ongoing logistics support. Thanks to Sello Matjee, Paige Ezzey, and Lesedi Moagi for their contributions to fieldwork during 2016–2019. The team also wishes to thank Dr Diego Gil, Dr Christina Riehl, and an anonymous reviewer for their valuable feedback that helped to improve the manuscript.
